# Case study: New onset of neuropsychiatric symptoms following switching to a dolutegravir regimen

**DOI:** 10.4102/sajpsychiatry.v28i0.1782

**Published:** 2022-03-29

**Authors:** Ahmed Badat, Karishma Lowton

**Affiliations:** 1 Department of Psychiatry, Faculty of Health Sciences, University of the Witwatersrand, Johannesburg, South Africa

**Keywords:** neuropsychiatric symptoms, dolutegravir, efavirenz, antiretrovirals, HIV

## Abstract

Although reports of neuropsychiatric side effects have been reported with efavirenz, these have been limited in comparison with regard to the now recommended dolutegravir regimens. We present a patient with new onset neuropsychiatric manifestations secondary to dolutegravir that resulted in significant physical injuries. The patient was initiated on risperidone for symptomatic control which was subsequently weaned and discontinued following reverting to an original efavirenz antiretroviral regimen, with resolution of neuropsychiatric symptoms. Neuropsychiatric side effects are increasingly noted with dolutegravir, and these should be monitored for on initiation and switching of treatment regimens.

## Background

In July 2019, the World Health Organization (WHO) recommended dolutegravir-based regimens as the preferred human immunodeficiency virus (HIV) treatment for all populations.^1^ Subsequently, the Department of Health (DoH) in South Africa published guidelines recommending initiation/switching to a combination of tenofovir, lamivudine and dolutegravir, following counselling on the benefits and risks of the regimen.^1^

These guidelines mention side-effects to the central nervous system (CNS), which are mild and self-limiting. However, there is increasing evidence that although occurring less frequently than efavirenz, neuropsychiatric symptoms have been reported on dolutegravir-based regimens.^2^ It is against this background that we present a case of neuropsychiatric symptoms, which developed in a patient following a change to a dolutegravir containing antiretroviral (ARV) regimen.

## Case presentation

Mr. X is a 32-year-old, married and employed male, who was referred to psychiatry from the trauma unit, following a fall from height. His highest level of education was Grade 12. This was his index presentation to psychiatry. Injuries sustained from the fall included a haemothorax, pericardial tear, anterior rib fracture and left humerus fracture. He is known with HIV since 2019 with a CD4 (cluster of differentiation 4) count of 205 and a viral load that was lower than detectable. According to collateral information, he had initially been stable on a combination of efavirenz, lamivudine and tenofovir, but was switched to dolutegravir, lamivudine and tenofovir a week prior to admission. There was no clearly documented reason for the change in treatment.

Symptoms surrounding his admission included sleep disturbances, dizziness and restlessness. These were worsening in severity over the week prior to the day of admission. He reported confusion and on discovering his wife having locked him in their home, he attempted to leave via a fire escape resulting in his fall from the fourth floor. According to his wife, his symptoms began after a change in the antiretroviral treatment (ART) regimen. She reported that he initially was not sleeping, talking alone and displayed aggression towards her on the day of admission. She had left the apartment to seek help after locking him inside.

During the psychiatric examination, he did not report any mood or psychotic symptoms; and there was no significant family history of psychiatric disorders and he had a good premorbid level of functioning. Of significance was the poor account of the circumstances leading to admission rendering him irrelevant at times during the interview. In view of recent surgeries and interventions, we opted to not commence antipsychotics and recommended managing any reversible causes of his presentation with a focus on pain control, hydration and adequate sleep.

On a follow-up interview, the patient was noted to become increasingly thought-disordered which hindered his engagement with the treating team. Risperidone 2 mg was commenced to which he responded well within a period of six days. At this stage, collateral information regarding the temporal relationship with his presentation and the change in antiretrovirals (ARVs) was confirmed and his ART was reverted to the original efavirenz based regimen.

The patient responded well to the change in ARVs and once discharged from the surgical team continued to follow up in the psychiatry outpatient department. Risperidone was subsequently weaned off and the patient was followed up for six months after stopping psychiatric treatment. There was no relapse of psychiatric symptoms, and he was discharged from psychiatry. He was given extensive psychoeducation on his diagnosis, reasons for the management plan and advice on returning to the hospital should relapse symptoms emerges.

## Literature review

Human immunodeficiency virus infection contributes significantly to the burden of disease in Southern Africa. As of 2019, the prevalence of HIV infection amongst the South African population was estimated to be 13.5% of the total population.^3^ The prevalence of mental illness amongst individuals infected with HIV is greater than that of the general population.^4^ Individuals with HIV infection may present with a range of psychiatric comorbidities, which may be pre-existing or arise following HIV infection. Neuropsychiatric complications are common in individuals with HIV and are related to a variety of factors ranging from direct effects of the virus on pre-existing conditions, personality vulnerabilities, substance use and disenfranchisement that individuals with HIV are, sometimes, subjected to.^5^ Mental illnesses that are commonly comorbid in individuals with HIV infection include schizophrenia, major depression, bipolar disorder, HIV-associated major neurocognitive disorder, post-traumatic stress disorder (PTSD), substance use disorders and delirium.^6^

Nucleoside Reverse Transcriptase Inhibitors (NRTIs) have been considered an integral part in combined ART, however they come with concerns of long-term toxicity and concerns of cross-resistance which has led to a developing interest in other treatment modalities.^7^ Dolutegravir is an integrase strand transfer inhibitor (INSTI) that offers intriguing pharmacological properties such as a prolonged intracellular half-life, making daily dosing feasible. Additionally, dolutegravir does not require meal related dosing and also shows a higher genetic barrier to resistance compared to other INSTIs.^8^ Other desirable characteristics of dolutegravir are fewer CNS reported symptoms, dosage and tablet size and fewer drug interactions.^8^ Dolutegravir in combination with abacavir and lamivudine showed a better safety profile and efficacy when compared to a combination of efavirenz, tenofovir and emtracitabine.^9^

The INSTIs are generally regarded as tolerable and safe.^10,11^ The most common adverse reactions reported with dolutegravir were mild to moderate nausea, diarrhoea and dizziness.^8^ The frequency of neuropsychiatric adverse effects reported in patients on dolutegravir occur less frequently than in patients on efavirenz^2^ with the most common being depression, insomnia, anxiety and abnormal dreams.^12,13,14^ The prevalence of specific neuropsychiatric adverse effects in patients on dolutegravir in South Africa is not known. Electronic medical records from the OPERA (Observational Pharmaco-Epidemiological Research and Analysis) database in the US were used to review the prevalence of neuropsychiatric adverse effects on 6314 patients who were receiving dolutegravir. From this sample, 5.0% reported insomnia, 4.8% reported anxiety and 0.1% reported suicidality following initiation on a dolutegravir-based regimen.^14^ A retrospective analysis of a cohort showed that the rate of discontinuation of dolutegravir as a result of neuropsychiatric adverse effects was higher when compared to other INSTIs, which has significant negative implications in managing HIV and comorbid psychiatric disorders.^15^ The CNS penetration effectiveness (CPE) score was defined by Letendre et al. as an indicator of an ARV drug’s ability to penetrate the CNS and suppress viral replication. It is based on the pharmacodynamic, pharmacokinetic and physiochemical properties of each drug.^16^ Antiretrovirals with higher CPE scores were thought to be ideal for patients with HIV associated neurocognitive symptoms, however the correlation between higher CPE scores and better neurocognitive outcomes have not been demonstrated in a randomised control trial.^17^ A review of CNS penetration of ARV drugs using pharmacokinetic data on which CNS penetrance inferences are based on showed that NRTIs have good CNS penetration, with the exception of tenofovir. In terms of non-nucleoside reverse transcriptase inhibitors (NNRTIs), efavirenz in its unbound form is considered to have high penetration into the CNS and is not actively cleared. The metabolites of efavirenz within the CNS are thought to result in neurotoxicity.^17^ In terms of INSTIs, dolutegravir has been shown to achieve therapeutic concentrations in the CNS.^18^

Even with the limited research available regarding the neuropsychiatric effects of dolutegravir, our patient reported symptoms as discussed in the research. Additionally, the temporal relationship of introduction and resolution of symptoms with the changes in ARV medication emphasised the importance of educating patients and monitoring for psychiatric symptoms accordingly.

Furthermore, training of HIV clinic staff in screening for psychiatric symptoms and provision of avenues for referral would be useful in managing these complications. Additional research may further influence guidelines and information found in such guidelines.
